# Changes in quality of life into adulthood after very preterm birth and/or very low birth weight in the Netherlands

**DOI:** 10.1186/1477-7525-11-51

**Published:** 2013-03-26

**Authors:** Afra van Lunenburg, Sylvia M van der Pal, Paula van Dommelen, Karin M van der Pal – de Bruin, Jack Bennebroek Gravenhorst, Gijsbert HW Verrips

**Affiliations:** 1 TNO, Child Health, Wassenaarseweg 56, Postbus 2215, Leiden, CE, 2301, The Netherlands

**Keywords:** Health-Related Quality of Life, Very preterm birth, Very low birth weight, Adulthood

## Abstract

**Background:**

It is important to know the impact of Very Preterm (VP) birth or Very Low Birth Weight (VLBW). The purpose of this study is to evaluate changes in Health-Related Quality of Life (HRQoL) of adults born VP or with a VLBW, between age 19 and age 28.

**Methods:**

The 1983 nationwide Dutch Project On Preterm and Small for gestational age infants (POPS) cohort of 1338 VP (gestational age <32 weeks) or VLBW (<1500 g) infants, was contacted to complete online questionnaires at age 28. In total, 33.8% of eligible participants completed the Health Utilities Index (HUI3), the London Handicap Scale (LHS) and the WHOQoL-BREF. Multiple imputation was applied to correct for missing data and non-response.

**Results:**

The mean HUI3 and LHS scores did not change significantly from age 19 to age 28. However, after multiple imputation, a significant, though not clinically relevant, increase of 0.02 on the overall HUI3 score was found. The mean HRQoL score measured with the HUI3 increased from 0.83 at age 19 to 0.85 at age 28. The lowest score on the WHOQoL was the psychological domain (74.4).

**Conclusions:**

Overall, no important changes in HRQoL between age 19 and age 28 were found in the POPS cohort. Psychological and emotional problems stand out, from which recommendation for interventions could be derived.

## Background

Over the last decades, the number of infants that survive a preterm birth has increased due to the progress in perinatal care. With the increase of surviving preterm infants and Very Low Birth Weight (VLBW) infants, another problem arises: the proportion of disabilities within this group of newborns also increases [1,2]. In the Netherlands the prevalence of live born preterms (22–37 weeks of gestation) is 7.3%. Within this group, 1.1% is born extremely preterm (22–32 weeks of gestation) and 1.0% has a VLBW (<1500 grams). Most of the infants with a VLBW are also born preterm [3]. Follow-up studies of those born with a VLBW show a wide variety of impairments [4], such as neurodevelopmental disabilities [5], blindness, deafness [6,7] and issues with growth [8] and learning [9]. A study of Tyson and Saigal (2005) shows that 16% of children with a VLBW had major neurosensory impairments, including cerebral palsy, deafness, and blindness. A quarter of the VLBW group had an IQ lower than 85 [2].

It is important to know the impact of Very Preterm (VP) birth or VLBW on health and Health-Related Quality of Life (HRQoL) to be able to provide the right (preventive) care in neonatal care units and later on in life. Next to the medical care, knowing the possible consequences on HRQoL can help professionals and parents in the decision making process of treating those born VP or with a VLBW. In the literature, there is no ultimate definition of the term HRQoL. Several studies choose the definition of health from the World Health Organization: ‘a state of complete physical, mental, and social well-being and not merely the absence of disease’ [9,10]. Others choose HRQoL to be defined as the value individuals assign to a particular health-state [9,11]. This study focuses more on this second definition. The Dutch ‘Project On Preterm and Small for gestational age infants’ (POPS) nationwide population based cohort of adults who were born VP or with a VLBW in 1983 [12], provides an unique possibility to study the long term effects of VP birth or VLBW on HRQoL of adults. Small for Gestational Age (SGA) is defined as a birth weight below the 10th percentile for gestational age and is associated with, for instance, increased neonatal complications [13]. The POPS study assessed three HRQoL questionnaires in adults aged 28 who were born VP or with a VLBW, giving a broad view on HRQoL. The transition into adulthood is an important stage of life, and important events such as finishing school and integration into work may affect HRQoL. Therefore, the purpose of this study is to evaluate changes in HRQoL of adults born VP or with a VLBW between age 19 and age 28.

## Methods

### Study population

**Figure 1:**
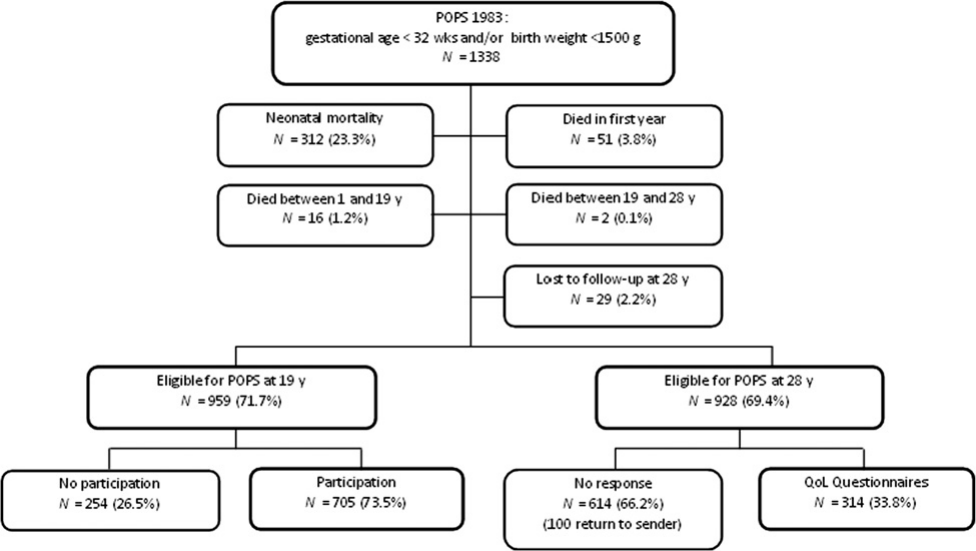
Flow chart inclusion of participants of the POPS study at age 28.

The POPS cohort included 1338 live-born, VP (gestational age <32 weeks) and/or VLBW (<1500 grams) infants born in the Netherlands in the year 1983 [12]. In total, 381 of these children did not survive to their 28th birthday, and 29 of them were lost to follow-up; 928 adults were eligible to participate in this follow-up study at 28 years of age (Figure 1).

### Assessment

In the year they would turn 28, individuals were invited to participate in the online study either through an email or a letter. Most participants filled in the questionnaire online (97.5%), a small group completed the questionnaire on paper on request (2.5%). Previously in the POPS cohort, data were collected at birth and ages two, five, nine, 10, 14 and 19 years [14]. In this present study we use these earlier collected data in addition to the data from the quality of life (QoL) questionnaires assessed at age 28.

### Ethical approval and informed consent

The medical ethics committee of the Leiden University Medical Center approved the study protocol. All participants sent in their written informed consent to participate in the study prior to the assessment.

### Data collection

HUI3 (Health Utilities Index, Mark 3) was used to assess HRQoL both on 19 and 28 years of age. HUI3 includes a summary of a comprehensive health status classification system [15], encompassing eight attributes of health: vision, hearing, speech, emotion, pain, ambulation, dexterity and cognition [16]. The level of functioning for each attribute is classified into five or six levels, ranging from ‘perfect function’ (level one) to ‘severe dysfunction’ (level five or six). With these levels of functioning, an eight-element health status vector can be established [16]. To provide a generic scale-score of HRQoL, where dead = 0 and perfect health = 1 [15], a Multi Attribute Utility (MAU) was calculated, as a generic score for the HUI questionnaire [16]. Because Dutch population reference scores are not available, this study uses the reference score for the Canadian population which is 0.85, and is the same for age 16–19 as for age 25–29, standard deviation is 0.18 and 0.17 respectively [22].

LHS (London Handicap Scale) was also used to assess HRQoL both on 19 and 28 years of age, focusing on the level of disability. LHS includes six dimensions of disability: mobility, physical independence (self-care), occupation (daily activities), social integration, orientation, and economic self-sufficiency; every dimension consist of a six-point hierarchical scale of disadvantages [17]. To provide the generic measure of disability (scale 0–100, where 100 is perfect health), a utility for LHS score was calculated based on the Dutch population preference index [18]. The six dimensions of disability are first recoded into a weighted score. Subsequently, the sum of these weighted scores for each dimension and 50.5 provides the LHS score 0–100.

At 28 years only, the WHOQoL-BREF (WHO Quality of Life instrument, short edition) was also assessed to determine HRQoL. The WHOQoL-BREF produces a quality of life profile divided into four domains: physical health, psychological, social relationships, and environment [19]. Domain scores from the WHOQoL-BREF were computed and transformed into weighted scores between 0–100, where 100 is perfect health.

### Analysis

The differences in characteristics of participants and non-participants were tested by chi-square tests in case of categorical variables or student’s t-tests in case of continuous variables. Characteristics that were tested: sex (male versus female), birth weight (in grams), origin (Dutch versus non-Dutch), educational level (low, middle or high), SES (low, middle or high), SGA versus appropriate for gestational age, maternal age at time of birth (in years), disabilities at five years of age (non, mild or severe), and disabilities at 10 years of age (non, mild or severe).

To adjust for missing values at age 19 and age 28 we applied multiple imputation [20] by using MICE (Multivariate Imputation by Chained Equations). This method “fills in” plausible values for the missing data, creating five imputed (completed) data sets. Predictive mean matching was used to create multiple imputations. The imputations are based on a model that uses information from the respondents and other variables to achieve optimal estimates. We pooled the results of the five imputed data sets to obtain data estimates, the precision of the estimates incorporates the uncertainty of the missing values. The original data set used for the multiple imputation contained the variables sex (male versus female), birth weight (in grams), origin (Dutch versus non-Dutch), educational level (low, middle or high), SES (low, middle or high), SGA versus appropriate for gestational age, maternal age at time of birth (in years), disabilities at five years of age (non, mild or severe), disabilities at 10 years of age (non, mild or severe), the items on HUI3 and LHS at age 19, and the items on HUI3, LHS and WHOQoL-BREF at age 28.

The difference in mean HRQoL scores between age 19 and age 28, both on HUI3 and LHS, was tested with a paired t-test, both on the original and imputed data. The mean WHOQoL score on the “Psychological” domain was tested against the mean score on “Social relationships” with a paired t-test. MAU can be categorized into four levels of disability: none, mild, moderate and severe [21]. Categorization of the MAU score and the eight single attributes score (X) was based on X=1 (none), 1>X>0.90 (mild), 0.90>X>0.70 (moderate) and X<0.70 (severe). Individual changes in MAU and attribute categories from age 19 to age 28 were classified into three categories: better (shift to a more favorable category), stable (no shift), and worse (shift to a less favorable category). MAU score was categorized to see if there was an important shift in disability from age 19 to age 28. To indicate how stable these scores are over time across the whole range of scores, pearson correlations were calculated. In addition, change in mean weighted scores on the eight attributes of HUI3 and the six dimensions of LHS were tested with a paired t-test from age 19 to age 28.

## Results

### Participant characteristics and non-response

Non-participants were more often male and non-Dutch, had a lower educational level and SES, and had more severe disabilities at age five than participants (Table 1).

**Table 1:** Characteristics of participants and non-participants at age 28

		**Participants n (%)**	**Non-participants**^**#**^**n (%)**	**p-value**
Sex*	Male	119 (38)	360 (59)	< 0.001
	Female	195 (62)	254 (41)	
Birth weight (grams)	<=1000	46 (15)	93 (15)	< 0.825
	1001-1250	89 (28)	169 (26)	
	1251-1500	111 (35)	258 (40)	
	>1500	68 (22)	123 (19)	
Origin*	Dutch	293 (94)	500 (82)	<0.001
	Non-Dutch	18 (6)	110 (18)	
Educational level (parents)*	Low	81 (26)	254 (46)	<0.001
	Middle	120 (38)	197 (36)	
	High	113 (36)	103 (18)	
SES (parents)*	Low	97 (31)	292 (48)	<0.001
	Middle	101 (32)	181 (30)	
	High	114 (37)	135 (22)	
Appropriate for gestational age	Yes	189 (60)	390 (64)	0.351
	No, small	124 (40)	224 (36)	
Maternal age at time of birth (years)	<20	11 (4)	54 (8)	0.065
	>=20 and	289 (92)	566 (88)	
	<36	14 (4)	23 (4)	
	>=36			
Disability at age 5*	None	89 (29)	119 (20)	<0.001
	Mild	215 (69)	427 (71)	
	Severe	8 (2)	53 (9)	
Disability at age 10	None	142 (51)	205 (50)	0.058
	Mild	119 (43)	149 (37)	
	Severe	18 (6)	52 (13)	

* p < 0.05.

# Survivors of the POPS cohort at age 28 who did not participate in this follow-up study at age 28.

### Overall (scale) scores HUI, LHS, WHOQoL

Table 2 shows that overall HRQoL on the HUI3, LHS and WHQoL were close to the optimal HRQoL score of 1 (HUI3) or 100 (LHS and WHOQoL-BREF). The WHOQoL “Psychological” domain score was lowest and significantly lower compared to the next-lowest WHOQoL domain score (“Social relationships”).

**Table 2:** Outcome in assessed 19 and 28-year-olds compared with outcome in all survivors at age 28

**Questionnaire**		**Assessed outcome n=314 Mean (sd)**	**MI**^**#**^**Outcome n=957 Mean (sd)**
Health Utilies Index 3 (Multi Attribute Utility)	19y	0.89 (0.16)	0.83 (0.22)
	28y	0.88 (0.16)	0.85 (0.20)
	Change 28y-19y	- 0.01 (0.15)	0.02 (0.17)*
London Handicap Scale (Utility)	19y	96.5 (8.3)	93.9 (12.4)
	28y	95.9 (8.0)	94.6 (9.8)
	Change 28y-19y	- 0.57 (7.5)	0.71 (9.0)
WHOQoL-BREF^$^ (Recoded into score 0–100)	Psychological	73.9 (14.7)*	74.4 (13.5)*
	Social	79.0 (17.3)	78.2 (16.9)
	Relationships	85.6 (12.9)	85.0 (12.8)
	Environment	85.8 (14.1)	85.8 (13.1)
	Physical health		

* p < 0.05.

^#^ MI: After multiple imputation.

^$^ Mean WHOQoL scores and standard deviations at age 28.

### Changes in HUI and LHS score from age 19 to age 28

Table 2 shows that both the mean HRQoL score measured with HUI3 and LHS did not change significantly from age 19 to age 28 in the original data. After multiple imputation, a significant increase was found in the mean MAU score from 0.83 at age 19 to 0.85 at age 28 (p=0.002). The mean individual MAU difference was 0.02 (sd=0.17; 95% CI −0.03 to −0.01). LHS showed no significant change after multiple imputation.

**Figure 2:**
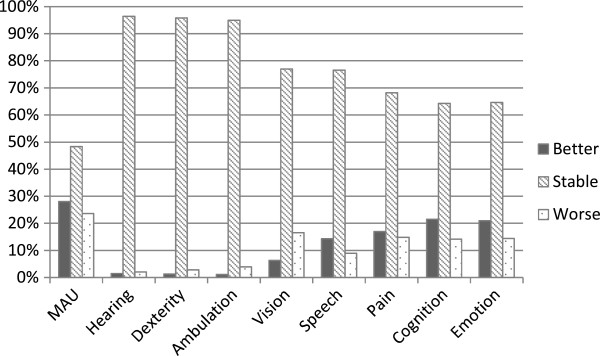
Distribution of MAU- and attribute change scores between ages 19 and 28 after multiple imputation.

Individual HUI scores, when divided into four levels of disability (none, mild, moderate, severe), improved in 28%, was stable in 48% and worsened within 24% of participants. Figure 2 shows this distribution of MAU-change scores and the change scores on its eight single attributes on HUI3 from age 19 to age 28 after multiple imputation; the physical attributes are more stable than the psychological attributes. Hearing and dexterity were both stable in 96% of participants, ambulation in 95%, and vision and speech were stable in 77% of participants. The psychological attributes pain, cognition and emotion show a bigger proportion of participants shifting to a better or worse category. Pain improved in 17% of participants, cognition in 22%, and emotion in 21%, respectively 15%, 14% and 14% shifted to a worse category. Pearson correlation scores over time on the eight single attributes on HUI3 show a positive correlation between cases at age 19 and cases at age 28: hearing r=0.468, dexterity r=0.916, ambulation r=0.906, vision r=0.478, speech r=0.332, pain r=0.390, cognition r=0.366, and emotion r=0.388. Dexterity and ambulation have the highest correlation, indicating the least change between the two ages. MAU score also showed a positive correlation (r=0.684).

**Figure 3:**
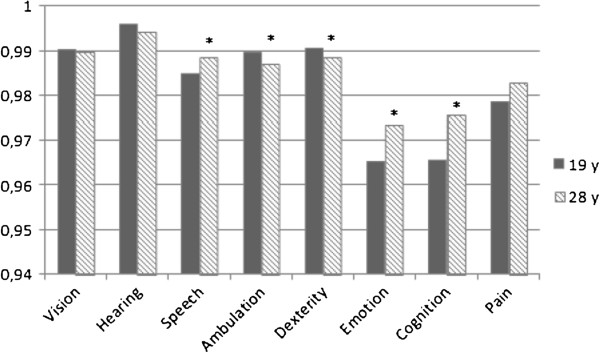
**Change in mean weighted scores on the eight attributes of HUI3**^**# **^**after multiple imputation.**

**Figure 4:**
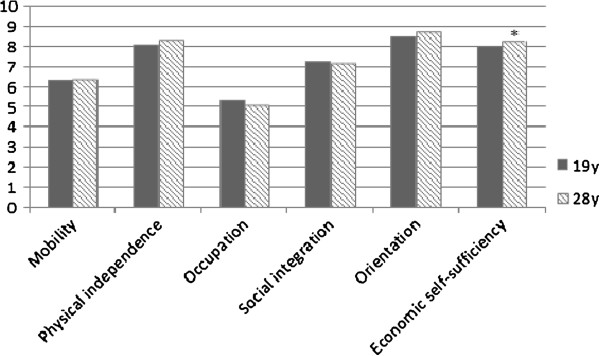
**Change in mean weighted scores on the six dimensions of LHS**^**# **^**after multiple imputation.**

Figure 3 shows the mean weighted scores on the eight attributes of HUI3 at age 19 and age 28 after multiple imputation. A significant decrease in mean weighted score on ambulation and dexterity from age 19 to age 28 is shown. Ambulation decreased from 0.9895 to 0.9869 and dexterity decreased from 0.9905 to 0.9884. Speech, emotion and cognition significantly improved from age 19 to age 28. The mean weighted score for speech increased from 0.9849 to 0.9885, for emotion from 0.9652 to 0.9735, and for cognition from 0.9656 to 0.9756. Figure 4 shows the mean weighted scores on the six dimensions on the LHS. The mean weighted score on economic self-sufficiency significantly increased from 8.00 at age 19 to 8.31 at age 28.

## Discussion

Overall, this study shows positive results in HRQoL scores for adults born VP or with a VLBW. Mean MAU score on the HUI3 decreased non-significantly from 0.89 at age 19 to 0.88 at age 28 in the original data. The imputed results show a significant increase of 0.02 in mean MAU score from 0.83 at age 19 to 0.85 at age 28. The imputed mean MAU scores were adjusted downward (indicating a lower HRQoL) compared to the original results, which can be explained by the higher number of disabilities in the imputed data. According to Horsman et al. [15], a difference of 0.03 is considered to be clinically important. Thus, no important changes in MAU score were found in the transition into adulthood in our population. A previous study by Verrips et al. [14] also showed no change in HUI3 scores from age 14 to age 19 within the POPS cohort. Unfortunately the POPS study has no matched control subjects, therefore a similar international cohort is used for comparison. A Canadian study [22] found similar results on HUI scores in young adults of 23 years of age: 0.85 (n=143 preterms) versus 0.88 controls (n=130) [9]. HUI3 reference score of HRQoL for adults aged 25–29 years is 0.85 (sd=0.17) [23]. However, due to cultural variations this comparison can not be interpreted as a main finding and the main aim of this study is to explore the change in HRQoL from age 19 to age 28. HRQoL score on the LHS did not change significantly from 96.5 (19y) to 95.9 (28y) in the original data, nor in the imputed data from 93.9 (19y) to 94.6 (28y). Saigal et al. [22] concluded that the young adults had adopted to their disabilities, which explains the high scores on HRQoL.

The WHOQoL scores at 28 years of age are high compared to the norm population: physical health 85.8 versus 78.8 (norm); social relationships 78.2 versus 72.3 (norm); and environment 85.0 versus 71.2 (norm) [24]. It is remarkable that the score on the “Psychological” domain is lower than in the norm population: respectively 74.4 versus 75.9 [24] and lower compared to the scores on the other WHOQoL domains. The facets that are incorporated with the psychological domain are: bodily image and appearance; negative feelings; positive feelings; self-esteem; spirituality / religion / personal beliefs; thinking, learning, memory and concentration [19]. Verrips et al. [14] already highlighted the relationship between psychological problems and HRQoL change from 14 to 19 years of age. The current study found the same effect in HRQoL change from 19 to 28 years of age. It seems that major health disabilities alone do not always predict one’s own perspective on HRQoL, as found in previous studies [5].

The change in individual MAU score from age 19 to age 28 was stable in 48%, improved in 28%, and worsened in 24% of participants. In comparison, from age 14 to age 19 individual MAU change score was stable in 45%, improved in 25%, and worsened in 30% of participants [14], nearly the same as in our study. There was a positive correlation over time across all the scores of the single eight attributes and the MAU score. Scores on dexterity and ambulation are high, almost one, indicating that these attributes are most stable over time. The psychological attributes, especially emotion and cognition, were less stable than the physical attributes. A part of the participants shifted to a worse category, but there was even a greater part that shifted to a better category, so there is hope for improvement. It might be of great importance to early monitor emotional well-being in this group. Interventions should be directed at dealing with (potential) disabilities within this group of children at teen age, to prevent later onset of emotional problems and to better manage pain. Especially because emotion, cognition and speech already improved significantly from age 19 to age 28, but overall score on the “Psychological” domain at age 28 were still significantly lower. A great emphasis in future research should be on the psychological problems that seem to be highly represented in adults born VP or with a VLBW. These psychological problems seem to influence changes of HRQoL during the transition into adulthood.

The non-response group in our study represents the same characteristics as found in the follow-up study at 19 years of age [25]: more often male, non-Dutch, lower educational level, lower SES and more severe disabilities. When these characteristics are not taken into account in the analyses, results may show an overestimation of the HRQoL of the POPS cohort. To correct for this selective dropout we applied multiple imputation. A limitation of this method though, is that the power gets artificially high and attention must be paid that two third of the data at age 28 is imputed rather than actually collected. On the other hand, POPS is a unique cohort with a very long follow-up from birth to 28 years of age, and multiple imputation can be based on the abundance of earlier collected data at birth and ages two, five, nine, 10, 14 and 19 years. Therefore the multiple imputation should give a good correction for non-response bias, resulting in a reliable outcome. Earlier data collected in the POPS study also included disability-status variables of the population. These variables can be used in the multiple imputation to correct for the selective dropout of those who might have been too disabled to be able to complete an internet survey.

## Conclusions

Overall, no important changes in HRQoL between age 19 and age 28 were found in our POPS cohort. Psychological and emotional problems are prominent and interventions should be directed at early detection, monitoring and managing these problems to decrease the negative impact on everyday life.

## Abbreviations

HUI3: Health utilities index mark 3; HRQoL: Health-related quality of life; LHS: London handicap scale; MAU: Multi attribute utility; POPS: Project on preterm and small for gestational age infants; SGA: Small for Gestational Age; VP: Very preterm; VLBW: Very low birth weight; WHOQoL-BREF: WHO quality of life instrument, short edition.

## Competing interests

The authors declare that they have no competing interests.

## Authors’ contributions

AL participated in the statistical analysis and drafted the manuscript. SP coordinated the study, participated in the statistical analysis and helped to draft the manuscript. PD, as statistical researcher, participated in the statistical analysis, especially the multiple imputation, and revised the manuscript. KP helped with her expertise as epidemiologist and revised the manuscript. JB participated in the statistical analysis, contributed with his knowledge on multiple imputation, and revised the manuscript. GV, as a quality of life expert, contributed to the HRQoL analyses and revised the manuscript. All authors read and approved the final manuscript.
